# Adaptive station selection incorporating observation data quality for UPD estimation

**DOI:** 10.1038/s41598-026-51937-6

**Published:** 2026-05-08

**Authors:** Shouzhou Gu, Long Xiao, Jinzhong Mi, Xuan Zhao, Yidong Fan, Bin Chu

**Affiliations:** 1 Chinese Academy of Surveying and Mapping, Beijing, 100036 China; 2 Beijing Fangshan Satellite Laser Ranging National Observation and Research Station, Beijing, 102406 China; 3 College of Geodesy and Geomatics, Shandong University of Science and Technology, Qingdao, 266590 Shandong China; 4 School of Surveying and Geoscience, Liaoning Technical University, Fuxin, 123000 China; 5 School of Electronic Information, Wuhan University, Wuhan, 430072 China; 6 Hunan Institute of Geomatics Sciences and Technology, Changsha, 410007 China

**Keywords:** Adaptive station selection, Dempster-Shafer (D-S) evidence theory, Dynamic grid, Marginal benefit, Observation data quality, Position dilution of precision (PDOP), Uncalibrated phase delay (UPD), Engineering, Mathematics and computing

## Abstract

To address the uneven spatial distribution and significant variations in observation data quality among multi-GNSS experiment (MGEX) stations, this paper proposes an adaptive station selection method (comprehensive adaptive site selection, CAS) for uncalibrated phase delay (UPD) estimation that incorporates observation data quality, thereby overcoming the limitations of traditional methods that neglect station geometry and data quality. A position dilution of precision (PDOP) and UPD error propagation model is developed. Using marginal benefit theory, the optimal number of stations is determined. A multi-indicator evaluation system based on Dempster-Shafer (D-S) evidence theory is established to assess data quality, enabling a dynamic grid algorithm that balances spatial geometry and data quality. The experiments are conducted using BeiDou‑3 navigation satellite system (BDS‑3) data. Experimental results demonstrate that the proposed method selects 80 optimal stations, accounting for only 30% of the global stations. The estimated Narrow-Lane (NL) UPD products achieve an accuracy better than 0.05 cycles, with a discrepancy of less than 0.002 cycles compared to the full-station solution, indicating comparable precision. Furthermore, the computational time is reduced by 54.1%.

**Supplementary Information:**

The online version contains supplementary material available at 10.1038/s41598-026-51937-6.

## Introduction

Precise point positioning (PPP) enables single receivers to achieve centimeter- to millimeter-level positioning accuracy. It has become a primary high-precision positioning method in the global navigation satellite system (GNSS) framework, with extensive applications in geodesy, disaster early warning, and meteorology. Ambiguity resolution (AR) enhances both the convergence speed and accuracy of PPP. High-precision uncalibrated phase delay (UPD) products are essential for achieving PPP-AR^1–3^. UPD estimation relies on observation data from globally distributed multi-GNSS experiment (MGEX) stations. However, factors such as the uneven spatial distribution of stations, variations in data quality, and residual systematic errors lead to challenges in current UPD estimation methodologies, including the lack of standardized station selection criteria and low computational efficiency^4^.

In recent years, numerous scholars have conducted in-depth research on UPD estimation theory, fostering the continuous advancement of this technology. The integer recovery clock (IRC) method was proposed, achieving for the first time the recovery of the integer nature of undifferenced ambiguities^5^. The Decoupled Satellite Clock (DSC) model was established, increasing the success rate of undifferenced ambiguity resolution to over 75%^6^. The first realization of BDS triple-frequency ambiguity resolution was achieved, significantly shortening the PPP convergence time to 15 min—a 40% reduction compared to dual-frequency solutions—by utilizing extra Wide-Lane (WL) combinations^7^. A hierarchical estimation method for triple-frequency UPDs was introduced, improving the accuracy of the ambiguity-fixed solutions by 60% in the horizontal component and 70% in the vertical component, ultimately achieving a positioning accuracy of 2.8 cm^8^. However, station selection methodologies have lagged behind the rapid evolution of UPD models, remaining largely empirical and based on listing experimental stations.There is a lack of unified standards, and the specific rationales for selection are seldom explicitly justified, which has become a key bottleneck constraining the robustness and cross-regional consistency of UPD products.

In other GNSS domains, station selection methodologies have developed a more systematic research framework. For precise orbit determination and Earth Rotation Parameter estimation, an optimized station selection model based on minimizing the Geometric Dilution of Precision (GDOP) value of the observation equations was proposed, significantly improving computational efficiency while maintaining orbit determination accuracy at 90% of the global solution^9^. Focusing on double-difference precise orbit determination, a station screening strategy that combines data preprocessing with quality assessments from the Toolkit for Epoch and Quality Check (TEQC) was introduced, thereby enhancing the reliability of orbit determination results by eliminating stations with poor data quality^10^. A method involving stochastic pulse parameters was employed to effectively compensate for deficiencies in existing dynamical models, thereby significantly improving the precision of BDS satellite orbit determination^11^. Concurrently, studies have also pointed out a saturation effect concerning the number of stations, indicating that beyond a certain point, increasing the number of stations offers limited improvement in accuracy while compromising computational efficiency^12–14^. Regarding data quality assessment, it has been highlighted that traditional single metrics are insufficient for comprehensively evaluating data reliability, leading to growing emphasis on multi-indicator fusion frameworks^15^. Based on Dempster-Shafer (D-S) evidence theory, a systematic grading of station data quality was implemented by constructing a three-level credibility model ("Good," "Medium," “Poor”)^16^. However, these studies are oriented towards orbit determination or positioning tasks. Their optimization objectives for station selection and quality indicator systems differ significantly from those required for UPD estimation and thus cannot be directly applied. UPD estimation imposes more stringent requirements on the uniformity of the geometric configuration of station combinations, the quality of observational data, and the stability of float ambiguity resolution. Particularly in the current context of a continuously growing yet highly unevenly distributed MGEX network, traditional methods like fixed-grid or empirically driven station selection are increasingly inadequate for achieving an optimal tripartite balance between accuracy, quality, and computational efficiency.

Addressing the issues mentioned above, this paper proposes an adaptive station selection method for UPD estimation that takes observational data quality into account. By establishing a Position Dilution of Precision (PDOP)-UPD error propagation model and introducing marginal benefit analysis, the optimal number of stations that balances precision gain and computational efficiency is determined. This is coupled with an observational data quality assessment based on D-S evidence theory, ultimately enabling adaptive station selection informed by data quality. The proposed method is designed for post-processing UPD product generation scenarios, performing quality evaluation and optimized station selection based on the full-day observational data. Experimental results demonstrate that this method significantly enhances both the accuracy of the estimated NL UPD products and the computational efficiency, offering a new perspective for station selection in server-side UPD estimation.

## Methodology

This section details the proposed adaptive station selection method for UPD estimation. The overall approach integrates an analysis of station geometry, a marginal benefit model for optimal station count, a D-S evidence theory-based quality assessment, and a dynamic selection algorithm. The rationale and formulation of each component are presented as follows.

## Determination of the optimal station number based on marginal benefit

### Impact of station distribution on UPD estimation

Raw carrier-phase ambiguities have short wavelengths and are susceptible to multipath and observation noise, making direct determination of their fractional parts difficult. A common strategy to overcome this is to first form WL ambiguities, which benefit from a significantly longer wavelength and are consequently easier to resolve to their correct integer values. These fixed WL integer ambiguities then serve as accurate constraints to aid in the resolution of the NL ambiguities when combined with the ionosphere-free carrier-phase observations^17–19^.

The calculation of float ionosphere-free ambiguities requires the construction of dual-frequency ionosphere-free carrier phase combination observations:

1 $$L_{IF} = \alpha_{1} L_{1} + \alpha_{2} L_{2}$$

where $\alpha_{1} = \frac{{f_{1}^{2} }}{{f_{1}^{2} - f_{2}^{2} }}$, $\alpha_{2} = - \frac{{f_{2}^{2} }}{{f_{1}^{2} - f_{2}^{2} }}$ Substituting the carrier phase observations, $L_{1}$ and $L_{2}$ into the Eq. (1) yields:

2 $$L_{IF,k} = \rho_{jk}^{i} + \varepsilon_{jk}^{i}$$

where $\rho_{jk}^{i} = \left[ {\left( {x_{k}^{i} - X_{{j_{k} }} } \right)^{2} + \left( {y_{k}^{i} - Y_{{j_{k} }} } \right)^{2} + \left( {z_{k}^{i} - Z_{jk} } \right)^{2} } \right]^{\frac{1}{2}}$ represents the geometric distance between satellite $i$ and station $j$ at epoch $k$. $(x,y,z)$ and $(X,Y,Z)$ denote the coordinates of the satellite and the observing station, respectively, in the Earth-Centered, Earth-Fixed (ECEF) coordinate system at the current epoch. $\varepsilon_{jk}^{i}$ represents the observation noise at the current epoch. Corrections for terms such as satellite clock errors, tropospheric delays, and Earth rotation parameters can typically be achieved by relying on precise estimations or utilizing existing precision products. Therefore, this paper primarily focuses on investigating the impact of the spatial geometric distribution of stations on the solution results.

Expanding the Eq. (2) using a first-order Taylor series:

3 $$\Delta L_{IF,k} = \frac{{\partial \rho_{k} }}{\partial x}\Delta x + \frac{{\partial \rho_{k} }}{\partial y}\Delta y + \frac{{\partial \rho_{k} }}{\partial z}\Delta z + \varepsilon_{k} = H_{k} {\mathbf{x}} + \varepsilon_{k}$$

where $H_{k}$ is the geometry matrix at epoch $k$, expressed as:

4 $$H_{k} = \left[ {\begin{array}{*{20}c} {\frac{{x_{k} - X_{k,1} }}{{r_{k,1} }}} & {\frac{{y_{k} - Y_{k,1} }}{{r_{k,1} }}} & {\frac{{z_{k} - Z_{k,1} }}{{r_{k,1} }}} \\ {\frac{{x_{k} - X_{k,2} }}{{r_{k,2} }}} & {\frac{{y_{k} - Y_{k,2} }}{{r_{k,2} }}} & {\frac{{z_{k} - Z_{k,2} }}{{r_{k,2} }}} \\ \vdots & \vdots & \vdots \\ {\frac{{x_{k} - X_{k,n} }}{{r_{k,n} }}} & {\frac{{y_{k} - Y_{k,n} }}{{r_{k,n} }}} & {\frac{{z_{k} - Z_{k,n} }}{{r_{k,n} }}} \\ \end{array} } \right]$$

where $r_{k,i}$ is the geometric distance from station $i$ to the satellite, $n$ is the number of stations, and $k$ is the observation epoch.

Applying the least squares principle, the corrections to the satellite positions are obtained as:

5 $$\Delta X = (H^{{\mathrm{T}}} H)^{ - 1} H^{{\mathrm{T}}} \Delta L_{IF,k}$$

Based on the aforementioned formulae, the error in the float ionosphere-free ambiguity induced by the station distribution can be expressed as:

6 $$\sigma_{r} = \sqrt {\sigma_{x}^{2} + \sigma_{y}^{2} + \sigma_{z}^{2} } = \sigma_{0} \sqrt {tr(H^{T} H)^{ - 1} }$$

7 $$Z_{PDOP} = \sqrt {tr(H^{T} H)^{ - 1} }$$

where tr denotes the trace of a matrix, which is the sum of its diagonal elements, and $Z_{PDOP}$ represents the PDOP value of the design matrix at that specific epoch. The PDOP value is determined by the geometry matrix $H$, which is defined by the relative positions of the global station network and the observable satellites. It directly quantifies the quality of the spatial geometry of the station-satellite configuration. A lower global PDOP value indicates a stronger, more favorable network geometry. This robust geometry effectively reduces the estimation error of the float ionosphere-free ambiguities, which is a prerequisite for high-quality UPD estimation. By enhancing the precision of the float solution, a low PDOP subsequently increases the success rates for fixing both WL and NL ambiguities. Therefore, the systematic analysis of PDOP provides a theoretical foundation for optimizing station selection, guiding the choice towards station combinations that offer superior spatial geometry.

### Marginal benefit analysis for optimal station count

In UPD estimation, based on the ionosphere-free combination model, the WL ambiguity is derived from the Hatch-Melbourne-Wübbena (HMW) combination, while the NL ambiguity is jointly resolved using the fixed integer WL ambiguity and the float ionosphere-free ambiguity^20^, as shown in Eqs. (8) and (9):

8 $$\hat{N}_{n,wl}^{m} = \frac{{L_{n,i}^{m} }}{{\lambda_{i} }} - \frac{{L_{n,j}^{m} }}{{\lambda_{j} }} - \frac{{\lambda_{j} P_{n,i}^{m} + \lambda_{i} P_{n,j}^{m} }}{{(\lambda_{j} + \lambda_{i} )\lambda_{wl} }} = N_{n,wl}^{m} + d_{n,wl} - d_{wl}^{m}$$

9 $$\hat{N}_{n,nl}^{m} = \frac{{\lambda_{lF} \hat{N}_{n,lF}^{m} }}{{\lambda_{nl} }} - \frac{{\lambda_{t} \cdot N_{n,wl}^{m} }}{{\lambda_{j} - \lambda_{i} }} = N_{n,nl}^{m} + d_{n,nl} - d_{nl}^{m}$$

where $\hat{N}$, $N$, and $\hat{N}_{lF}^{{}}$ represent the float ambiguity, integer ambiguity, and float ionosphere-free ambiguity, respectively; $L$ and $P$ denote the carrier phase and pseudorange observations, respectively; $m$ and $n$ refer to the satellite and receiver ends, respectively; $d_{n}$ and $d^{m}$ are the UPDs at the receiver and satellite ends, respectively. As shown in Eqs. (8) and (9), the float ambiguity can be expressed as a combination of the integer ambiguity and the UPDs at the receiver and satellite ends. The virtual observation equation is constructed as follows:

10 $$R_{n}^{m} = d_{n} - d^{m}$$

where $R_{n}^{m}$ represents the fractional part of the float ambiguity.

According to the fundamental principle of least squares adjustment, solving for the UPD parameters requires that the system of observation equations contains a sufficient number of linearly independent equations. Specifically, the number of linearly independent equations must exceed the total number of unknown UPD parameters to be estimated^21^.The key parameters involved in UPD estimation and their quantitative relationships are detailed in Table 1.

**Table 1:** Statistical table of parameters for UPD estimation.

Parameter	Quantity/number
Satellites	*s*
Receiver-end UPD	*k-1*
Satellite-end UPD	*s*
Average visible satellites per Epoch per Station	*u*

To ensure the estimability of the NL UPDs, the rank condition of the system of observation equations must be satisfied. Based on the redundancy requirements for parameter estimation, the following inequality constraint must be established:

11 $$k \times u \ge (k - 1) + s + d$$

where $d$ denotes the required redundancy for ambiguity resolution.

Based on the average number of satellites observable per epoch from actual stations, the minimum number of stations required can be determined parametrically using the above equation. Assuming a globally uniform distribution of stations, the solution obtained from the inequality can satisfy the preliminary requirements for UPD estimation.

Marginal benefit (MB) is a core concept in economics, referring to the additional benefit gained from adding one unit of a particular input, holding all other inputs constant. Its fundamental principle is the Law of Diminishing Marginal Returns (LDMR): as the input of one factor is continuously increased, its marginal benefit typically experiences an initial increase, followed by a decrease, eventually approaching zero or even turning negative^22^.

The relationship between the number of stations and the PDOP value conforms to the law of diminishing marginal returns. This manifests specifically as follows: when the number of stations is small, an increase in their number leads to a significant reduction in the PDOP value, representing a stage of high marginal benefit. As the number of stations continues to increase, the degree of PDOP reduction gradually diminishes until it approaches zero. Therefore, by collecting data on the number of stations and their corresponding PDOP values, marginal benefit analysis can be employed to determine the optimal number of stations. This method evaluates the improvement in PDOP value resulting from adding each new station to identify the inflection point where the marginal benefit decreases significantly. The analysis is conducted using the following two quantitative metrics:

The absolute marginal benefit indicator measures the absolute reduction in the PDOP value caused by adding a new station. It is calculated using the following formula:

12 $$\Delta Z_{PDOP,abs} = Z_{PDOP,w} - Z_{PDOP,w + 1}$$

where $Z_{PDOP,w}$ is the PDOP value corresponding to $w$ stations. When the absolute marginal benefit falls below a threshold $\Delta Z_{PDOP,abs} < T_{abs}$, adding a new station provides no significant improvement.

The relative marginal benefit indicator measures the relative rate of change in the PDOP value induced by adding a new station. It is calculated using the following formula:

13 $$\Delta Z_{PDOP,rel} = \frac{{Z_{PDOP,w} - Z_{PDOP,w + 1} }}{{Z_{PDOP,w} }}$$

where the search for the optimal number of stations is terminated when the relative marginal benefit falls below a threshold $\Delta Z_{PDOP,rel} < T_{rel}$.

The final optimal number of stations is determined by comprehensively analyzing the changing trends of these two benefit curves. The specific thresholds $T_{abs}$ and $T_{rel}$ are determined through statistical analysis of actual experimental data.

## Observational data quality assessment based on D-S evidence theory

The quality of station observational data is a critical factor affecting the accuracy of UPD estimation, influenced by elements such as station hardware performance and environmental interference. Although employing high-performance receivers can mitigate certain errors to some extent, practical observation processes still contend with issues like multipath effects and turbulence-induced atmospheric deviations, leading to systematic biases in the station observational data.

To address these factors, this paper employs a D-S evidence theory-based comprehensive assessment method^16^ using four indicators: multipath error, signal-to-noise ratio, cycle slip ratio, and data completeness rate. The specific methodology is as follows.

Firstly, a three-level indicator threshold framework (“Good”, “Medium”, “Poor”) is established, and basic probability assignments are determined using membership functions. Membership functions range from 0 to 1, reflecting the degree of association between the data and a specific characteristic, with a higher value indicating a stronger association. Increasing-type membership functions are used for positive indicators, while decreasing-type functions are used for negative indicators. The GNSS data automated quality checking software Anubis is used to process each station’s data and obtain the corresponding quality evaluation indicators. Among these, multipath error is a negative indicator, employing a decreasing-type membership function; the other three are positive indicators, employing increasing-type membership functions. The specific functions are as follows:

14 $$\mu_{{\mathrm{1,max}}} = \left\{ {\begin{array}{*{20}c} {1,r \ge a} \\ {0,r{\text{ < a}}} \\ \end{array} } \right. ,\mu_{1,\min } = \left\{ {\begin{array}{*{20}c} {1,r \le a} \\ {0,r> a} \\ \end{array} } \right.$$

15 $$\mu_{2} = \left\{ {\begin{array}{*{20}c} {\frac{r - b}{{a - b}},b \le r < a} \\ {\frac{c - r}{{c - a}},a \le r < c} \\ {0,r < b,r \ge c} \\ \end{array} } \right.$$

16 $$\mu_{{\mathrm{3,max}}} = \left\{ {\begin{array}{*{20}c} {1,r \le b} \\ {\frac{a - r}{{a - b}},b < r \le a} \\ {0,r> a} \\ \end{array} } \right.,\mu_{{\mathrm{3,min}}} = \left\{ {\begin{array}{*{20}c} {1,r \ge c} \\ {\frac{r - a}{{c - a}},a < r \le c} \\ {0,r \le a} \\ \end{array} } \right.$$

where a, b, c are the thresholds of the membership function’s identification framework, and $r$ represents the respective quality evaluation indicator. These thresholds are determined based on prior knowledge and expert experience^23^.The specific thresholds for the membership functions of each observational data type are listed in Table 2.

**Table 2:** Thresholds for evaluating the quality of observational data.

Parameter	Multipath error (cm)	Signal-to-noise ratio (dBHz)	Cycle slip ratio (%)	Data completeness rate (%)
a	0.3	35	800	85
b	0.5	40	1 000	95
c	0.7	45	1 200	100

After obtaining the membership functions for each indicator, normalization is applied to these functions to derive the Basic Probability Assignment (BPA) function. Subsequently, by integrating the Belief function, Plausibility function, and an evaluation function, a weighted comprehensive evaluation model is established. This model accurately reflects the contribution degree of each indicator to the final assessment result through the configuration of differentiated weights, ultimately achieving a quantitative evaluation of the overall credibility of the observational data. The specific comprehensive evaluation model is as follows:

17 $$F = \sum\limits_{i = 1}^{n} {\omega_{i} \cdot f_{{(x_{i} )}} }$$

where $f_{{(x_{i} )}}$ is the evaluation function for each sample data point, and $\omega_{i}$ is the corresponding weight coefficient for the indicator. The prior weights for each indicator are determined based on prior knowledge and expert experience^23^, as shown in Table 3. The discrete level classification and ranking-based selection strategy ensure that moderate variations in membership thresholds and weights do not alter the relative station rankings or the final UPD estimation precision.

**Table 3:** Comprehensive weighting scheme.

Parameter	Multipath error	Signal-to-noise ratio	Cycle slip ratio	Data completeness rate
Weight	0.25	0.125	0.125	0.5

By applying Eq. (17), the observational data quality of stations can be assessed. This process integrates multiple evaluation indicators through comprehensive weighting to establish a scientific quality grading standard. This provides reliable data quality assurance for UPD estimation, thereby contributing to enhanced accuracy and reliability of the UPD estimation results.

## Adaptive grid-based station selection method

### Fixed grid selection method

The fixed grid station selection method is realized through spatial partitioning based on a latitude–longitude grid. The selection process is as follows: first, the required number of stations is determined; then, the corresponding number of latitude–longitude grid cells is calculated based on the number of stations; finally, the division of the latitude–longitude grid is completed in both the longitudinal and latitudinal directions. This enables the spatial partitioning of global stations. The formula for determining the number of stations via the latitude–longitude grid is as follows:

18 $$N = \left\lceil {\frac{360}{p} \times \frac{180}{p}} \right\rceil$$

where $p$ is the angular dimension (in degrees) of the grid cell, $\left\lceil {} \right\rceil$ denotes the ceiling function, and $N$ represents the required number of stations.

The fixed grid method, which employs a uniform latitude–longitude grid, presents the following issues: (1) The global distribution of stations exhibits significant spatial non-uniformity, with dense concentrations in regions like Europe and North America, and sparse coverage in Africa, South America, and parts of Asia. Using a fixed grid size results in some grid cells containing numerous stations, while others remain empty. (2) When the target number of stations is a small prime number, achieving an optimal grid partition becomes mathematically challenging. (3) The method selects stations based solely on geographical distribution, failing to adequately incorporate the quality of the observational data. Consequently, the selection process is susceptible to significant subjective influence.

### Proposed comprehensive adaptive site selection (CAS) method incorporating data quality

To address the limitations of the fixed grid method, this paper proposes a density-adaptive grid-based station selection method that incorporates observational data quality, termed the comprehensive adaptive site selection (CAS). This method establishes a comprehensive observational data quality assessment system. Based on the target number of stations and the actual global station density, it dynamically determines the optimal latitude–longitude grid size, prioritizing stations with high-quality observational data. The aim is to strike a balance between global coverage completeness and regional selection refinement, ensuring the objectivity and reliability of the station selection results.

First, the observational data quality assessment method based on D-S evidence theory is used to conduct a comprehensive evaluation of each station’s data quality. The evaluation outputs two data quality scores: stations with “Good” or “Medium” quality are represented by an F_total_ value and are suitable for UPD estimation; stations with “Poor” quality are represented by an F_c_ value and are unsuitable for UPD estimation. To ensure the reliability of the data from stations participating in UPD estimation, a quality threshold F_0_ is set, eliminating all substandard stations where $F_{c}> F_{0}$, thereby forming the candidate station set.

Unlike the fixed grid size, this method’s grid division process starts by determining an initial grid size based on the required number of stations $N_{{{\mathrm{target}}}}$. This initial size must ensure that the total number of grid cells $N_{grid}$ meets the selection requirements for the target number of stations, adhering to the following specific constraint:

19 $$N_{grid} = \left[ {\frac{360}{g} \times \frac{180}{g}} \right] \ge 1.2 \times N_{{{\mathrm{target}}}} ,g_{\min } \le g \le g_{\max }$$

where $g$ represents the grid size. This condition ensures a sufficient number of grid cells to accommodate the target number of stations. If the condition is not met, the grid size $g$ is iteratively reduced (g→0.9g) until the condition is satisfied or the minimum grid sizes are reached, where $g_{\min }$ is 5°and $g_{\max }$ is 30°.

After determining the initial grid size, the grid size $g$ is dynamically optimized based on the actual spatial density $\rho$ of the stations: in high-density regions ($\rho> \rho_{0}$), a finer grid is used to enhance selection precision, while in low-density regions ($\rho < \rho_{0}$), a coarser grid is employed to ensure global coverage, thereby achieving spatially adaptive station optimization. Here, the station density $\rho$ is defined as $\rho = \frac{N}{S}$, with units of stations per 10,000 km^2^, where $N$ is the number of stations within the current grid cell and $S$ is the area of the grid cell. The specific adaptive formula for the grid size is as follows:

20 $$g = g_{\min } + (g_{\max } - g_{\min } ) \cdot \left( {1 - \min \left( {1,\frac{\rho }{{\rho_{0} }}} \right)} \right)$$

where $\rho_{0}$ is the specified baseline density value.

After the grid division is completed, a differentiated selection strategy is applied based on the number of qualified stations within each grid cell: For grid cells containing only one station with qualified observational data, that station is selected directly. For grid cells containing multiple stations with qualified observational data, the station with the highest data quality evaluation score is preferentially selected.

This method achieves optimal station selection through the synergistic interaction of the density-adaptive grid and the multi-dimensional evaluation model. By automatically adjusting the grid size according to the actual station density, it yields a station combination that meets the requirements of UPD estimation and possesses a reasonable distribution. The method mitigates the subjective biases inherent in traditional approaches, thereby enhancing the reliability of the station selection results.

## Experiments and results

To comprehensively validate the effectiveness of the proposed adaptive station selection methodology, a series of experiments were conducted using real-world MGEX data. The evaluation focuses on three key aspects: 1) verifying the determination of the optimal station number through marginal benefit analysis; 2) assessing the performance of the data quality evaluation model based on D-S evidence theory; and 3) comparing the overall accuracy and computational efficiency of the proposed adaptive method against traditional approaches.

### Experimental data and setup

This study employs the optimization of station selection for post-processing Beidou-3 Global Navigation Satellite System (BDS-3) Uncalibrated Phase Delay (UPD) estimation as a case study. Using in-house developed software on an Ubuntu virtual platform, we processed observational data from 266 global Multi-GNSS Experiment (MGEX) tracking stations/International GNSS Service (IGS) reference stations over a continuous 7-day period from Day of Year (DOY) 71 to 77, 2025. The station selection process was executed offline once per day. The specific processing strategies for UPD estimation are detailed in Table 4. It should be noted that the computation times reported in this section refer only to the UPD estimation process and do not include the offline station selection overhead, which is relatively small given that the selected station set remains reasonably stable across consecutive days.

**Table 4:** UPD estimation strategies.

Category	Processing strategy
True coordinates	IGS weekly solution files
Sampling interval	30 s
Cut-off elevation angle	10°
Satellite antenna	IGS antenna correction products
Ephemeris	Wuhan University mixed-constellation rapid/final precise ephemerides
Clock products	Wuhan University mixed-constellation rapid/final precise clock products
Relativistic effect	Model correction
Tide corrections	Solid Earth tide, ocean tide loading, pole tide
Tropospheric model	Dry delay model correction, wet delay estimated via random walk
Ionospheric model	Ionosphere-free linear combination

### Determination of the optimal station number via marginal benefit analysis

**Fig. 1:**
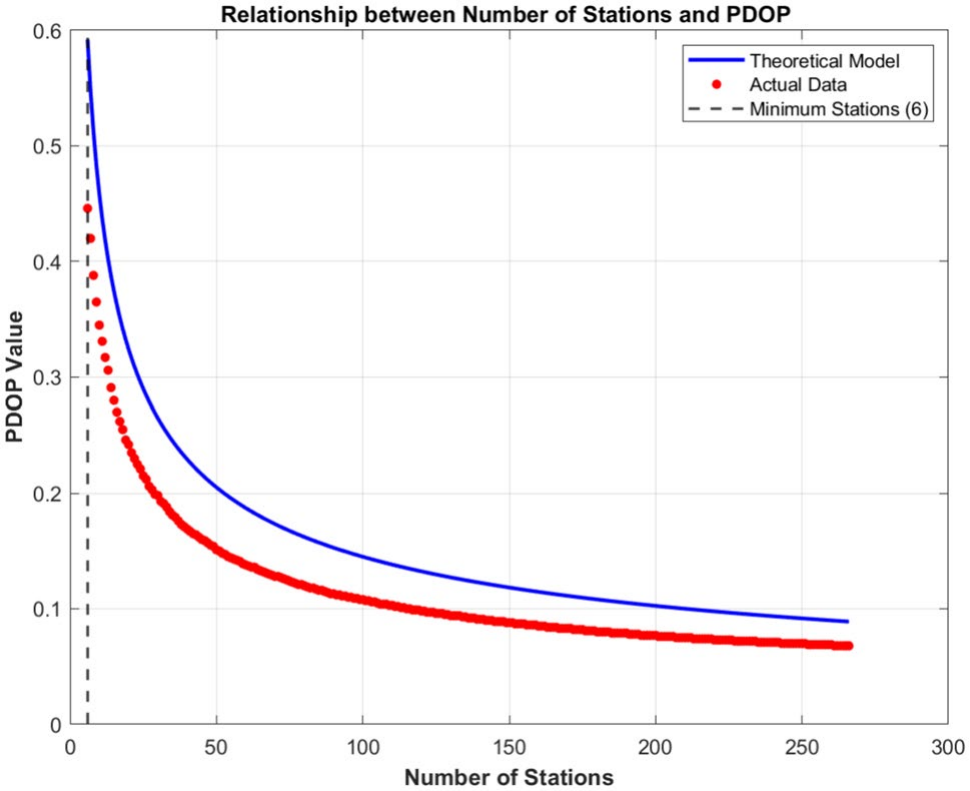
Relationship between PDOP values and the number of stations.

Analysis of the BDS-3 observational data from the stations indicates that an average of 10 to 12 satellites are observable per epoch. According to the parametric inequality, when using BDS-3 satellites for the experiment, a minimum of 6 globally and uniformly distributed stations is required to meet the fundamental prerequisites for UPD estimation.After determining the minimum number of stations, the PDOP values were calculated through the following procedure: (1) Station coordinates were extracted from the IGS weekly solution files; (2) Visible satellites were identified based on a cut-off elevation angle of 10°; (3) The geometry matrix H was constructed according to the formula; (4) The corresponding PDOP values for different numbers of stations were computed. Starting from the minimum number of stations, the number was gradually increased, and the corresponding PDOP values were calculated. A graph depicting the relationship between the actual PDOP values, the theoretical PDOP values, and the increasing number of stations was plotted. The PDOP-versus-station-number curve presented in this study is based on the PDOP values calculated for the global station network combination. This calculation ensures that after each station addition, the geometric configuration formed by the entire set of candidate stations and the visible satellites is re-evaluated. The relationship between PDOP value and the number of stations is shown in Fig. 1.

As shown in Fig. 1 both the actual and theoretical PDOP values exhibit a convergent trend as the number of stations increases. Specifically, when the number of stations increases gradually from the minimum of 6, the PDOP value decreases rapidly. The most significant reduction, with an improvement rate of 63% in the station geometry, occurs within the range of 20 to 40 stations. When the number of stations exceeds 70, the change in PDOP value stabilizes. Beyond this point, adding further stations yields only limited improvement to the geometric configuration.

**Fig. 2:**
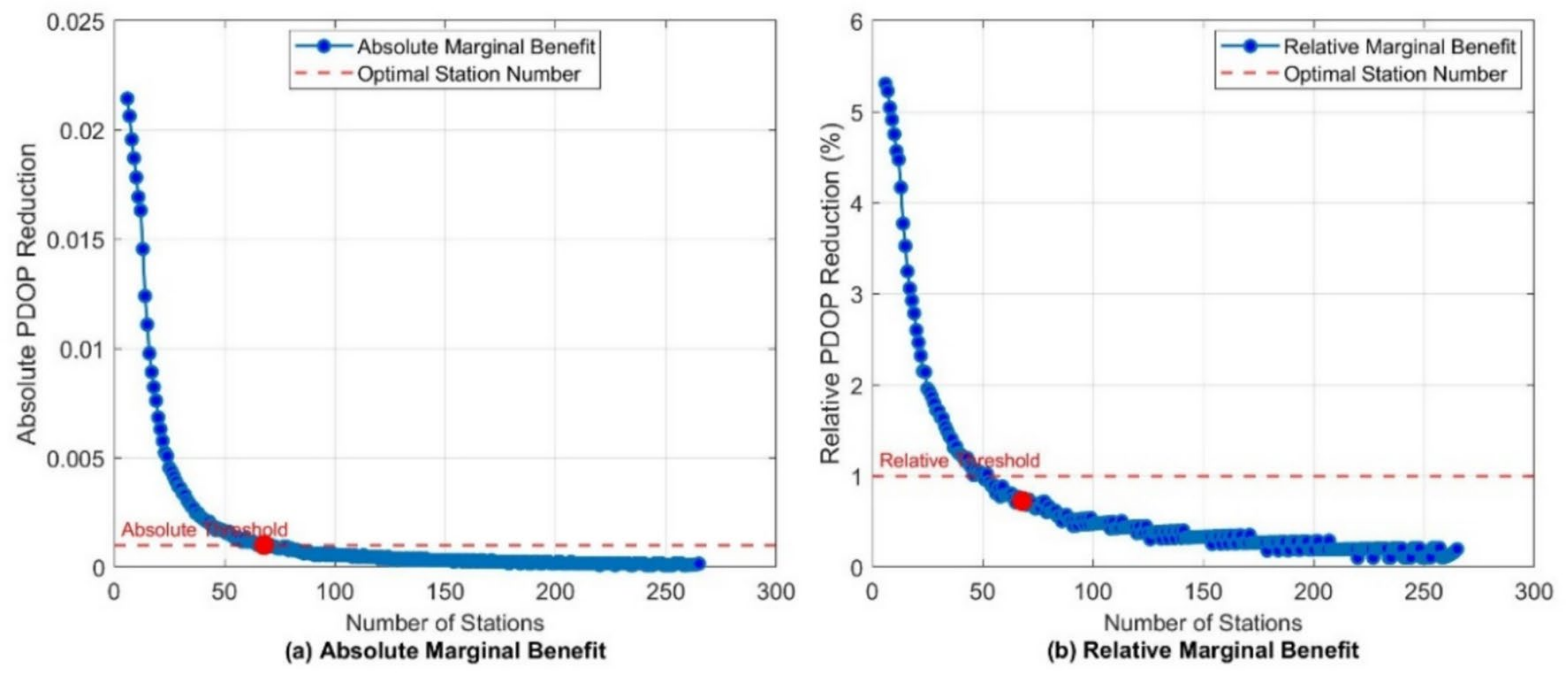
Marginal benefit curves: (**a**) absolute marginal benefit; (**b**) relative marginal benefit.

Based on the observed pattern of PDOP values, the marginal benefit analysis method proposed in Sect. 2.1.2 was applied. According to the characteristics of the actual data, the absolute benefit threshold was set to 0.001 and the relative benefit threshold was set to 0.01. Figure 2 presents the marginal benefit curves: (a) absolute marginal benefit and (b) relative marginal benefit as functions of the number of stations. Both curves drop below the preset thresholds when the station count reaches 70, indicating saturation of the geometric improvement.

Figure 2 indicates that when the number of stations increases to 70, both the absolute and relative changes in the PDOP value fall below the set thresholds. This signifies that the spatial geometry of the station network has approached its theoretical saturation point. Consequently, 70 stations are deemed sufficient to meet the fundamental geometric configuration requirements for UPD estimation. However, considering system fault tolerance and the actual uneven spatial distribution of stations, this experiment adds 10 extra stations to the base of 70, thereby enhancing the reliability of the UPD products in complex real-world data environments. Subsequent experiments will be conducted based on this configuration of 80 stations.

### Data quality assessment based on D-S evidence theory

The Anubis software was used to calculate the observational data quality indicators—namely multipath error, signal-to-noise ratio, cycle slip ratio, and data completeness rate—for each station, following the quality assessment methodology outlined in Sect. 2.2. Subsequently, the membership functions corresponding to these four indicators were normalized to generate the Basic Probability Assignment (BPA) function for each. Finally, a weighted comprehensive evaluation was performed by integrating the Belief function, Plausibility function, and evaluation function, based on the indicator weights presented in Table 3, to compute the final quality score F_total_ for the stations. Table 5 displays the quality indicators for selected stations on DOY 75, 2025.

**Table 5:** Statistical table of data indicators for selected stations.

Station	Multipath error (cm)	Signal-to-noise ratio (dBHz)	Cycle slip ratio (%)	Data completeness rate (%)
ACRG	43.9	45.12	273	92.02
ALRC	26.0	44.64	22905	98.71
BREW	37.0	38.35	412	99.08
CUSV	27.2	45.91	784	96.69
FAIR	44.4	39.96	226	97.08
HERS	0.0	43.40	646	99.80
KOKB	40.3	38.56	524	98.78

**Fig. 3:**
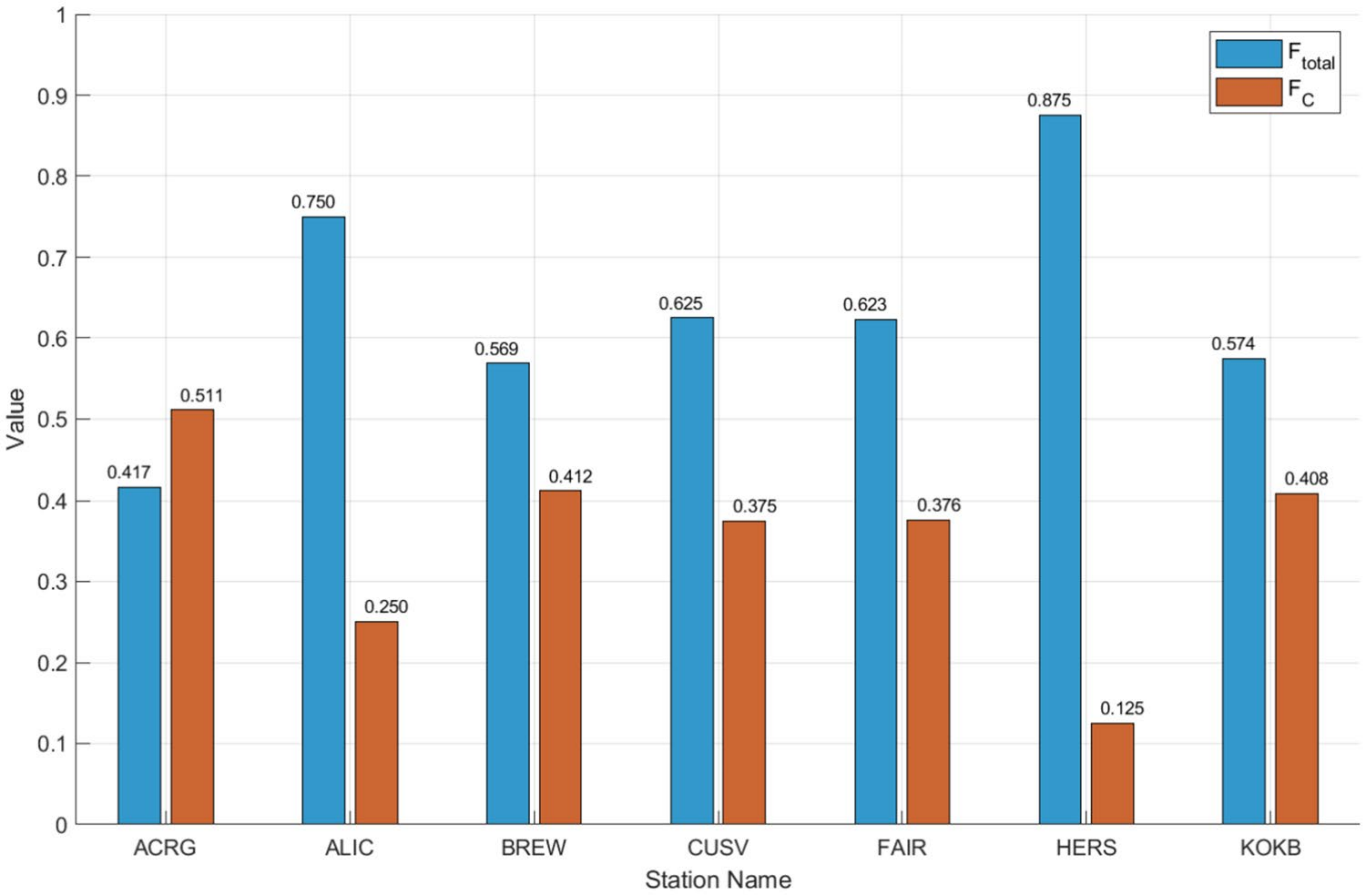
Quality assessment results of observation data for selected stations (DOY 75, 2025).

Figure 3 shows the calculated data quality scores F_total_ and Fc for each station based on the indicators in Table 5. A higher F_total_ indicates better observation data quality, while a higher F_c_ indicates poorer quality. Among the stations, HERS exhibits the best data quality, whereas ACRG shows the poorest. In the figure, F_total_ represents the data quality score for stations suitable for UPD estimation, encompassing data of “Good” and “Medium” quality levels. A higher F_total_ value indicates better observational data quality for the corresponding station. F_c_ represents the data quality score for stations unsuitable for UPD estimation, where a higher F_c_ value indicates poorer data quality. Both scores have a value range of 0 to 1.

As shown in Fig. 3, the station credibility assessment results align with the membership functions established in Sect. 2.2. Multipath error is a negative indicator; a lower value corresponds to a higher F_total_ score, indicating better station observational data quality. Conversely, signal-to-noise ratio, cycle slip ratio, and data completeness rate are positive indicators; higher values correspond to a higher F_total_ score, indicating better data quality. Among the stations shown, HERS station exhibits the best observational data quality, while ACRG station exhibits the poorest.

### Comprehensive analysis of CAS

Based on the CAS method for UPD estimation considering observational data quality proposed in Sect. 2.3, the optimal station combination was determined for the quality-assessed stations using the following steps: (1) Stations with F_c_ > 0.4 were eliminated to ensure data reliability; (2) The global grid size was initially set to 29.97°based on the optimal station count of 80, and then dynamically adjusted according to the actual station density; (3) Differentiated station selection was performed based on the number of stations within each grid cell, finalizing a station combination that balances both geometric configuration and data quality.

Given the high stability of WL UPDs, this study focuses on NL UPD estimation results, which are more sensitive to station selection methods. Using the experimental results from DOY 75, 2025, as an example, the performance differences in UPD estimation resulting from four distinct station selection methods are systematically evaluated. This is achieved by sequentially presenting the following for each method: the spatial distribution map of the selected stations, the time series of the estimated NL UPDs, the standard deviation plot, and the residual distribution plot. The four methods compared are: the Fixed Grid Selection (FGS), the Fixed Grid Selection considering observational data Quality (Q-FGS), the Comprehensive Adaptive Selection (CAS), and the All-Stations Solution (ASS).

**Fig. 4:**
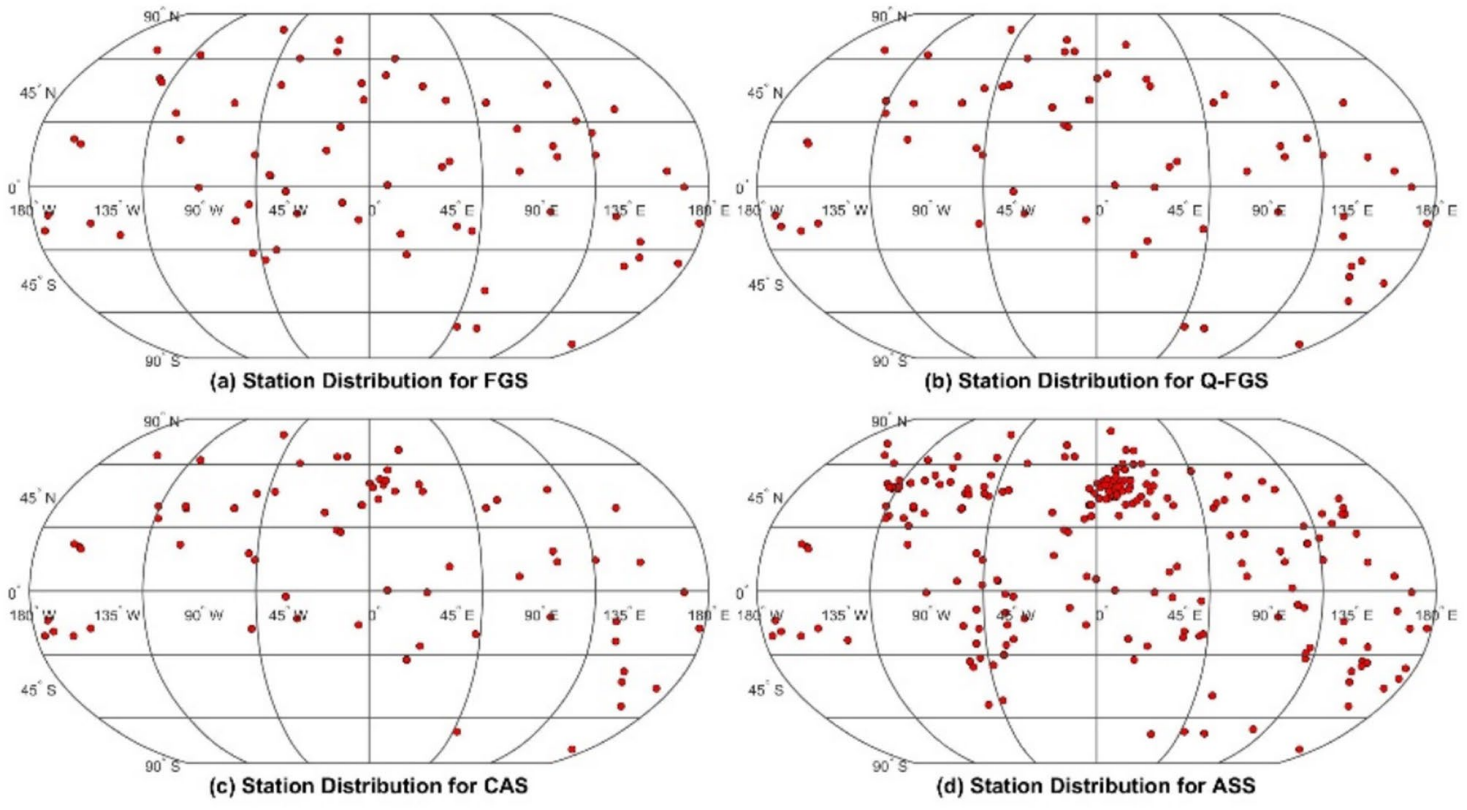
Spatial distribution of stations selected by different methods.

Figure 4 compares the spatial distributions of stations selected by the four methods. Compared to the All-Stations method, the other three methods significantly reduce the number of stations. Notably, CAS yields a denser distribution in high-density regions such as Europe, demonstrating effective density-adaptive selection. Maps were generated using MATLAB R2021b (version 9.11) and the Mapping Toolbox (version 5.2) (https://www.mathworks.com/). No third‑party geographic data were used.

**Fig. 5:**
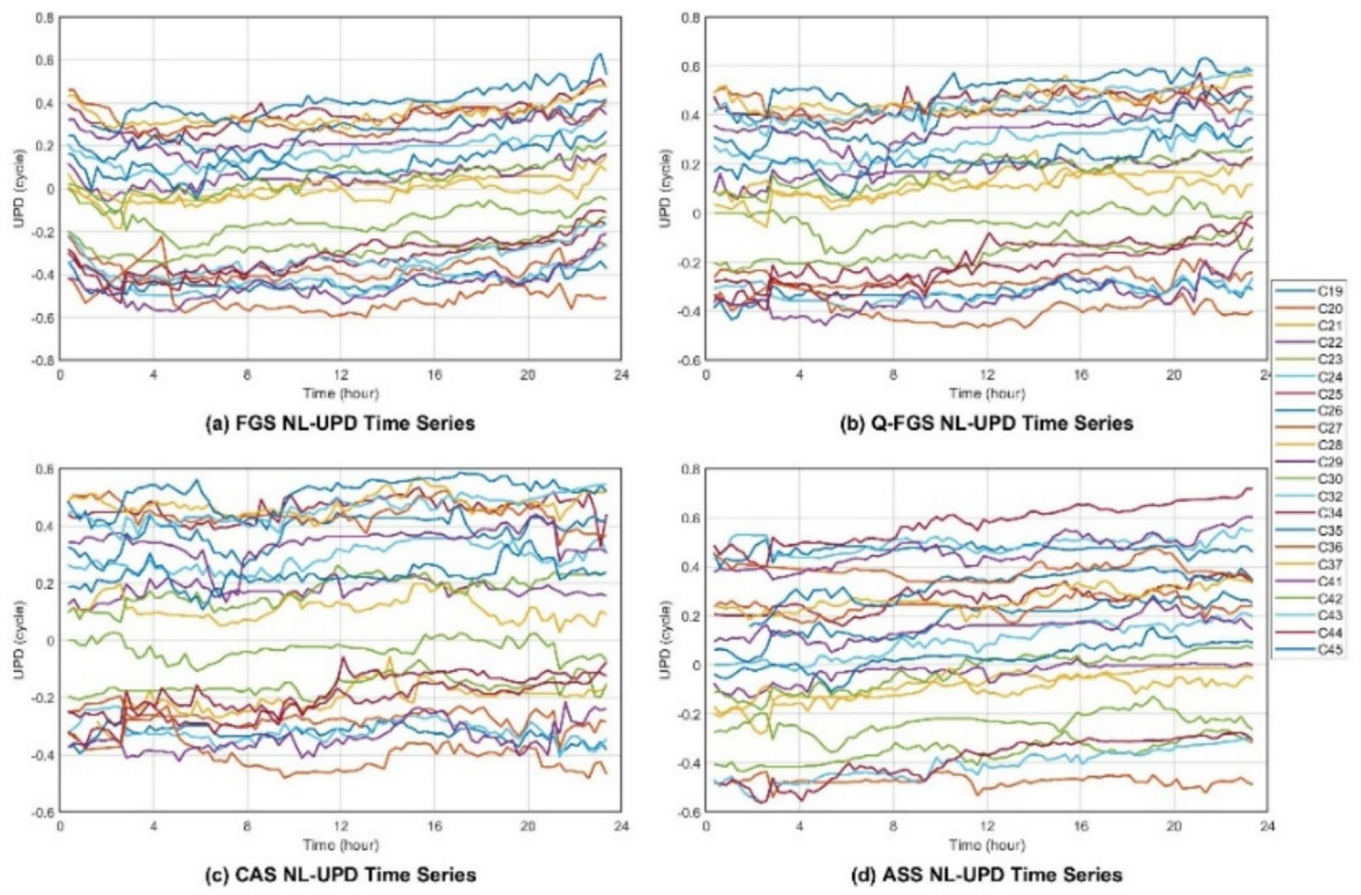
Time series of NL UPDs estimated by different station selection methods.

Figure 5 displays the time series of NL UPDs estimated using the different station selection methods. The UPD series from the Fixed Grid method exhibits the most significant fluctuations, with a maximum variation amplitude reaching 0.21 cycles. The series from the Fixed Grid method considering data quality shows some improvement, yet its maximum variation amplitude remains as high as 0.18 cycles. While the All-Stations method demonstrates good stability, it incorporates data fluctuations from all stations. In contrast, CAS achieves superior temporal stability, with a maximum variation of merely 0.12 cycles. This represents reductions of 43% and 33% compared to the first two methods, respectively, while maintaining accuracy comparable to the All-Stations method.

**Fig. 6:**
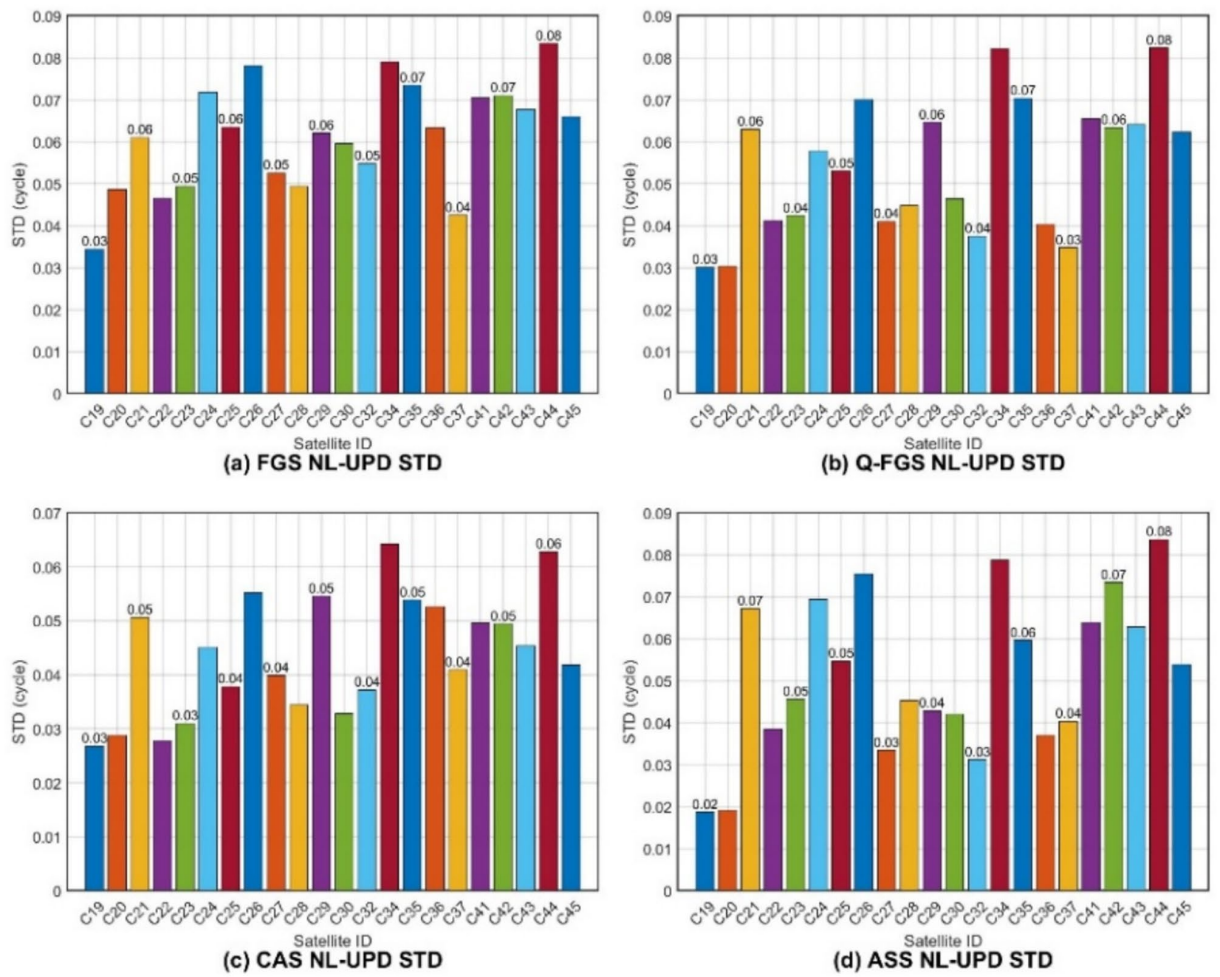
Comparison of standard deviations of NL UPDs.

Figure 6 compares the standard deviations of the NL UPDs estimated by the different station selection methods. The Fixed Grid method yields the highest dispersion in the results, with a range of standard deviations reaching 0.059 cycles. The Fixed Grid method considering data quality shows improved accuracy, reducing the range to 0.042 cycles. While the range of standard deviations for the All-Stations method is similar to that of CAS, it incurs a significantly higher computational burden. CAS achieves optimal precision control, with a range of only 0.038 cycles. This represents a 35% reduction compared to the standard Fixed Grid method, demonstrating that by comprehensively considering both geometric configuration and data quality, it effectively enhances the consistency of UPD estimation.

**Fig. 7:**
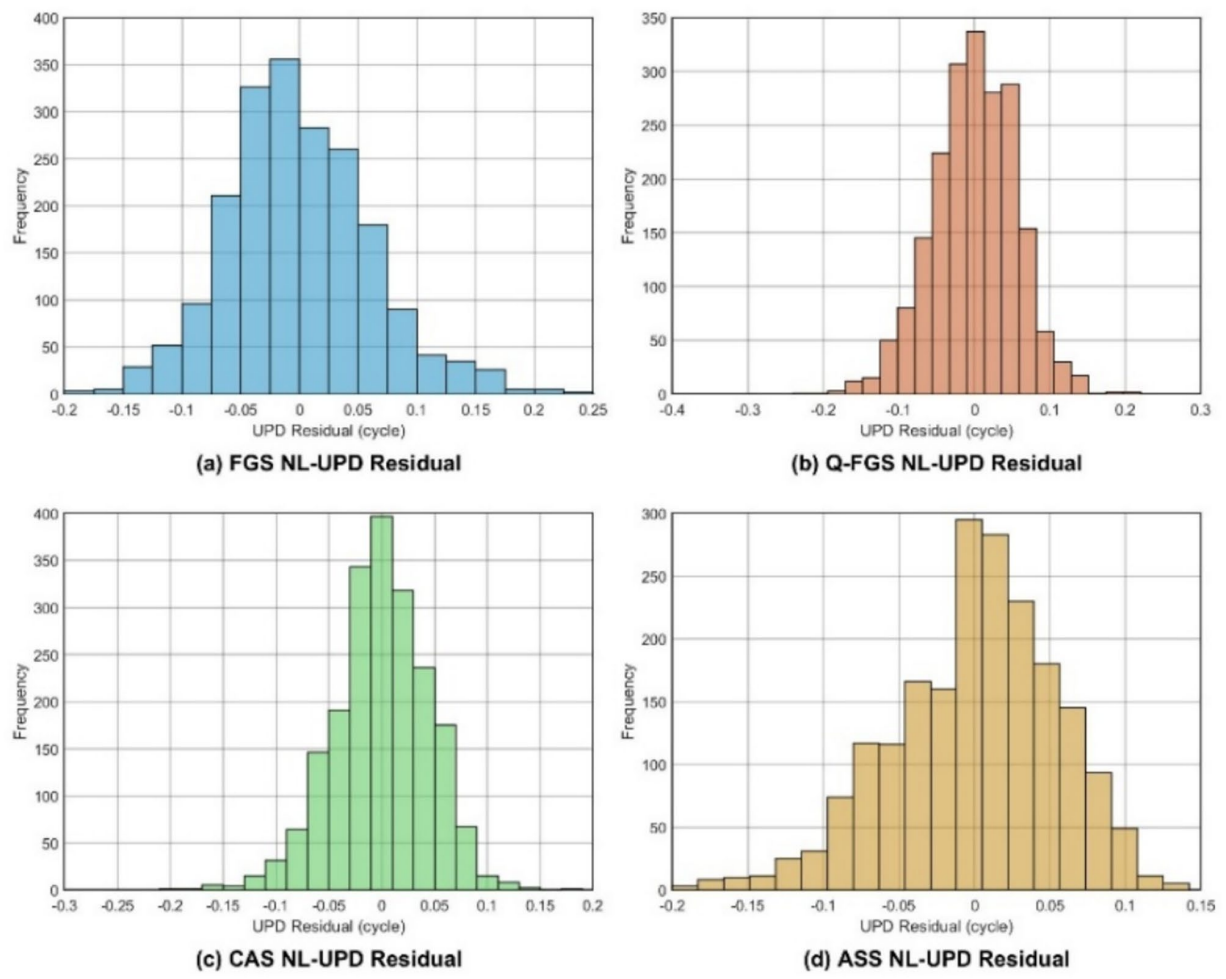
Residual distribution of NL UPDs.

Figure 7 shows the residual distributions of the NL UPDs obtained from the different station selection methods. The residual distribution from the Fixed Grid method exhibits a pronounced.

Right-skew, indicating the presence of systematic bias. The distribution from the Fixed Grid method considering data quality shows some improvement but remains asymmetric. The All-Stations method, due to its inclusion of lower-quality data, yields a more dispersed residual distribution. In contrast, the residual distribution from CAS most closely approximates the ideal normal distribution, characterized by the highest peak and the best symmetry, demonstrating its comprehensive advantage in error control.

To verify the general applicability of the comprehensively optimized station selection method, Table 6 provides further statistics on the NL UPD estimation results over five consecutive days (DOY 71–77). A comparative analysis is conducted across four dimensions: average standard deviation, residual Root Mean Square (RMS), PDOP value, and computation time. The specific statistical results are presented in Table 6.

**Table 6:** Comparison of NL UPD estimation results.

Doy	Method	Average STD (cycle)	Residual RMS (cycle)	PDOP value	Computation time (s)
71	FGS	0.054	0.054	0.119	2055
	Q-FGS	0.037	0.038	0.120	2194
	CAS	0.030	0.031	0.120	2203
	ASS	0.029	0.030	0.066	4915
72	FGS	0.071	0.072	0.119	1986
	Q-FGS	0.044	0.045	0.120	2123
	CAS	0.037	0.037	0.120	2096
	ASS	0.038	0.040	0.067	4874
73	FGS	0.067	0.068	0.122	2049
	Q-FGS	0.046	0.046	0.122	2186
	CAS	0.045	0.045	0.122	2217
	ASS	0.049	0.050	0.068	4866
74	FGS	0.081	0.081	0.119	1954
	Q-FGS	0.047	0.048	0.120	2265
	CAS	0.046	0.046	0.120	2241
	ASS	0.046	0.047	0.067	4913
75	FGS	0.067	0.068	0.119	2016
	Q-FGS	0.054	0.056	0.119	2247
	CAS	0.044	0.045	0.120	2263
	ASS	0.052	0.055	0.065	4925
76	FGS	0.064	0.064	0.122	2123
	Q-FGS	0.047	0.049	0.122	2254
	CAS	0.039	0.040	0.123	2234
	ASS	0.041	0.042	0.067	4876
77	FGS	0.051	0.052	0.119	2083
	Q-FGS	0.045	0.046	0.120	2263
	CAS	0.033	0.033	0.121	2194
	ASS	0.037	0.037	0.066	4837

As can be seen from Table 6, compared with the Fixed Grid method, CAS reduces the average standard deviation from 0.0647 cycles to 0.0391 cycles, a reduction of 39.6%, and reduces the residual RMS from 0.0656 cycles to 0.0398 cycles, a reduction of 39.3%, while achieving comparable PDOP values. Compared with the Fixed Grid method considering observational data quality, CAS reduces the average standard deviation from 0.0457 cycles to 0.0391 cycles, a reduction of 14.4%, and reduces the residual RMS from 0.0468 cycles to 0.0398 cycles, a reduction of 15.0%, while maintaining comparable PDOP values and largely comparable computation time. The smaller performance gap on DOY 73 and 74 is due to the larger candidate pool (about 150 stations) on those days, which reduces the relative advantage of CAS. CAS is most beneficial when the number of qualified stations is limited. Compared with the All-Stations method, CAS shows differences in average standard deviation of less than 0.002 cycles and in residual RMS of less than 0.003 cycles, indicating comparable accuracy between the two; meanwhile, the average computation time is reduced from 4889.8 to 2243.2 s, resulting in a 54.1% improvement in computational efficiency.

## Conclusion

To address the limitations in current UPD estimation methods, particularly the neglect of global station distribution non-uniformity and variations in data quality, this paper proposes the comprehensive adaptive site selection (CAS) method. The experimental results lead to the following conclusions:

First, based on marginal benefit analysis, the theoretical saturation point for satisfying geometric configuration requirements was identified at approximately 70 stations. To enhance robustness and fault tolerance in practical applications, the optimal number of stations was comprehensively determined to be 80. Using only 30% of the total available stations, this strategy achieved accuracy comparable to the all-station solution while reducing computation time by 54.1%.

Second, by jointly considering observational data quality and spatial distribution characteristics, CAS ensures global spatial uniformity in the selection results while preferentially incorporating stations with higher data quality.

Third, in terms of NL UPD estimation performance, CAS reduced the average standard deviation of NL UPD products by 39.6% and the residual RMS by 39.3% compared to the Fixed Grid method. Compared to the Fixed Grid method with data quality consideration, it further reduced the average standard deviation by 14.4% and the residual RMS by 15.0%. Notably, the method achieved slightly improved accuracy in NL UPD estimation (with differences less than 0.003 cycles) compared to the All-Stations method, while improving computational efficiency by 54.1%.

This study focuses on post-processing UPD product estimation, where both quality assessment and station selection are based on full-day data. Future work will explore the extension of this method to real-time UPD services, which will require the introduction of sliding-window statistics for instantaneous quality monitoring and the use of lightweight optimization algorithms to enhance the efficiency of station selection and solution estimation. In addition, the applicability of the proposed method to other GNSS constellations (GPS and Galileo) will be investigated in future studies.

## Supplementary Information

Supplementary Information.

## Data Availability

All other data generated or analysed during this study are included in this published article. The raw GNSS observation datasets used in this study are publicly available from the IGS Data Center at Wuhan University at http://www.igs.gnsswhu.cn/. No registration or application is required for access.
