# Supplementary material for: Adaptive station selection incorporating observation data quality for UPD estimation
Source: Sci Rep. 2026 May 8;16:21151. doi: 10.1038/s41598-026-51937-6 (PMC13342249; doi:10.1038/s41598-026-51937-6)
Supplement: Supplementary file 1 — Supplementary Information. [file 41598_2026_51937_MOESM1_ESM.docx]

Table A1. Performance comparison of different station selection methods over DOY 63–77, 2025 (15 days).

| Doy | Method | Average STD (cycle) | Residual RMS (cycle) | PDOP Value | Computation Time (s) |
| --- | --- | --- | --- | --- | --- |
| 63 | FGS | 0.062 | 0.063 | 0.121 | 2011 |
|  | Q-FGS | 0.042 | 0.043 | 0.121 | 2158 |
|  | CAS | 0.035 | 0.036 | 0.122 | 2176 |
|  | ASS | 0.034 | 0.035 | 0.065 | 4852 |
| 64 | FGS | 0.075 | 0.076 | 0.118 | 1969 |
|  | Q-FGS | 0.049 | 0.050 | 0.119 | 2214 |
|  | CAS | 0.041 | 0.042 | 0.119 | 2209 |
|  | ASS | 0.044 | 0.045 | 0.068 | 4907 |
| 65 | FGS | 0.058 | 0.059 | 0.122 | 2074 |
|  | Q-FGS | 0.039 | 0.040 | 0.122 | 2173 |
|  | CAS | 0.032 | 0.032 | 0.123 | 2188 |
|  | ASS | 0.036 | 0.037 | 0.066 | 4845 |
| 66 | FGS | 0.079 | 0.080 | 0.119 | 1947 |
|  | Q-FGS | 0.051 | 0.052 | 0.120 | 2259 |
|  | CAS | 0.047 | 0.048 | 0.120 | 2245 |
|  | ASS | 0.050 | 0.051 | 0.067 | 4931 |
| 67 | FGS | 0.065 | 0.066 | 0.120 | 2038 |
|  | Q-FGS | 0.045 | 0.046 | 0.120 | 2236 |
|  | CAS | 0.038 | 0.039 | 0.121 | 2227 |
|  | ASS | 0.047 | 0.048 | 0.065 | 4889 |
| 68 | FGS | 0.055 | 0.056 | 0.118 | 2092 |
|  | Q-FGS | 0.040 | 0.041 | 0.119 | 2165 |
|  | CAS | 0.034 | 0.034 | 0.119 | 2199 |
|  | ASS | 0.032 | 0.033 | 0.066 | 4828 |
| 69 | FGS | 0.072 | 0.073 | 0.122 | 1995 |
|  | Q-FGS | 0.048 | 0.049 | 0.123 | 2248 |
|  | CAS | 0.044 | 0.045 | 0.123 | 2256 |
|  | ASS | 0.049 | 0.050 | 0.068 | 4918 |
| 70 | FGS | 0.060 | 0.060 | 0.120 | 2059 |
|  | Q-FGS | 0.043 | 0.044 | 0.121 | 2221 |
|  | CAS | 0.036 | 0.037 | 0.121 | 2213 |
|  | ASS | 0.039 | 0.040 | 0.067 | 4864 |
| 71 | FGS | 0.054 | 0.054 | 0.119 | 2055 |
|  | Q-FGS | 0.037 | 0.038 | 0.120 | 2194 |
|  | CAS | 0.030 | 0.031 | 0.120 | 2203 |
|  | ASS | 0.029 | 0.030 | 0.066 | 4915 |
| 72 | FGS | 0.071 | 0.072 | 0.119 | 1986 |
|  | Q-FGS | 0.044 | 0.045 | 0.120 | 2123 |
|  | CAS | 0.037 | 0.037 | 0.120 | 2096 |
|  | ASS | 0.038 | 0.040 | 0.067 | 4874 |
| 73 | FGS | 0.067 | 0.068 | 0.122 | 2049 |
|  | Q-FGS | 0.046 | 0.046 | 0.122 | 2186 |
|  | CAS | 0.045 | 0.045 | 0.122 | 2217 |
|  | ASS | 0.049 | 0.050 | 0.068 | 4866 |
| 74 | FGS | 0.081 | 0.081 | 0.119 | 1954 |
|  | Q-FGS | 0.047 | 0.048 | 0.120 | 2265 |
|  | CAS | 0.046 | 0.046 | 0.120 | 2241 |
|  | ASS | 0.046 | 0.047 | 0.067 | 4913 |
| 75 | FGS | 0.067 | 0.068 | 0.119 | 2016 |
|  | Q-FGS | 0.054 | 0.056 | 0.119 | 2247 |
|  | CAS | 0.044 | 0.045 | 0.120 | 2263 |
|  | ASS | 0.052 | 0.055 | 0.065 | 4925 |
| 76 | FGS | 0.064 | 0.064 | 0.122 | 2123 |
|  | Q-FGS | 0.047 | 0.049 | 0.122 | 2254 |
|  | CAS | 0.039 | 0.040 | 0.123 | 2234 |
|  | ASS | 0.041 | 0.042 | 0.067 | 4876 |
| 77 | FGS | 0.051 | 0.052 | 0.119 | 2083 |
|  | Q-FGS | 0.045 | 0.046 | 0.120 | 2263 |
|  | CAS | 0.033 | 0.033 | 0.121 | 2194 |
|  | ASS | 0.037 | 0.037 | 0.066 | 4837 |
